# The Diminishing Importance of Primary Site Identification in Cancer of Unknown Primary: A Canadian Single-Center Experience

**DOI:** 10.3389/fonc.2021.634563

**Published:** 2021-03-03

**Authors:** Boaz Wong, Michael M. Vickers, Paul Wheatley-Price

**Affiliations:** ^1^ Department of Biochemistry, Microbiology and Immunology, Faculty of Medicine, University of Ottawa, Ottawa, ON, Canada; ^2^ Cancer Therapeutics Program, Ottawa Hospital Research Institute, Ottawa, ON, Canada; ^3^ Division of Medical Oncology, The Ottawa Hospital Cancer Centre, Ottawa, ON, Canada

**Keywords:** cancer of unknown primary, cancer epidemiology, patient prognosis, cancer diagnostics, favorable subtype, retrospective analysis

## Abstract

**Background:**

Cancer of unknown primary (CUP) describes patients with metastatic disease without an identified primary tumor site. Successful diagnosis and treatment of these patients remains difficult. Published guidelines on CUP have highlighted “favorable” subtype groups. We investigated a series of CUP patients to review adherence to guidelines, and identification of primary cancers or “favorable” subtypes.

**Methods:**

Patients with histologically confirmed CUP at an academic institution from 2012 to 2018 were identified. Patient demographics, tumor presentation, diagnostic work-up and treatment information were retrospectively collected from electronic data records for descriptive analysis and compared to published clinical guidelines. The primary endpoint was the proportion of patients where the primary site was identified. Multivariable logistic regression models were used to identify factors associated with primary site identification. Kaplan-Meier survival curves were used to determine factors associated with poorer OS.

**Results:**

Three hundred and five patients were included with a median follow-up time of 4.3 months. Primary tumor sites were identified in 109 patients (37.5%), which was most commonly lung cancer (33%). Statistical analyses did not identify any demographic or initial presentation factors associated with identifying the primary or not. More diagnostic tests did not increase the likelihood of primary site identification (*P=0.44*). Patients with an identified primary did not have longer OS than other patients (median 5.2 months vs. 4.7 months, *P=0.47*). 57 patients (18.7%) who had a defined “favorable” subtype experienced superior OS (36.6 months vs. 3.8 months; *P<0.0001*). Further, patients with good prognostic status who followed published treatment guidelines had longer OS (17.6 months vs. 13.2 months; *P=0.04*).

**Conclusions:**

CUP remains a difficult cancer to diagnose and treat. These results suggest identifying the primary has less impact than anticipated, but particular efforts to identify patients with “favorable” subtypes of CUP is important prognostically.

## Introduction

Cancer of unknown primary site (CUP) describes the diagnosis of a metastatic cancer where the location of the primary tumor is unable to be identified following thorough medical investigation (1). Despite the steady decline in CUP incidence from 5% since the 1980s to around 2% of all new invasive cancer diagnoses, the prognosis for patients remains poor and is the fourth most common cause of cancer death (2–4). From 2000 to 2005, 3,564 new cases of CUP were diagnosed in Ontario, Canada (5).

A thorough diagnosis and work-up of the primary tumor site is paramount for directing treatment options, especially given the emergence of targeted therapies. At baseline, published guidelines by the National Comprehensive Cancer Network (NCCN), European Society of Medical Oncology (ESMO) and Spanish Society of Medical Oncology (SEOM) require a thorough medical history, physical examination, basic blood and biochemistry analyses, imaging, immunohistochemical analysis of biopsies and other specific tests where necessary (6–8). The ultimate aim when investigating CUP remains to try and identify the primary tumor site in order to optimize treatment plans according to other published guidelines. Median overall survival (OS) times for a patient with a diagnosed primary tumor site in Ontario are significantly longer than CUP patients (median, 11.9 months vs. 1.9 months) (9). Additionally, not only can proper workup suggest primary tumor sites, but also identify patients who may fall into a “favorable” subtype with a known treatment regimen (**Table 1**). Examples of favorable subtypes include neuroendocrine tumors, isolated axillary nodal metastases in females or non-supraclavicular cervical squamous cell carcinomas. These CUP patients make up 20% of all cases and have well-defined treatment regimens towards dramatically improved survival outcomes (10, 11).

**Table 1:** Table outlining common “favorable” clinical subtypes and recommended treatment compiled from the European Society of Medical Oncology (ESMO) and Spanish Society for Medical Oncology (SEOM) (6, 8).

Clinical subtype	Recommended treatment
**Females with isolated axillary adenopathy**	As per stages II–III breast cancer
**Females with peritoneal adenocarcinomatosis**	As per stage III ovarian cancer
**Poorly-differentiated neuroendocrine carcinoma**	Platinum + Etoposide
**Well-differentiated neuroendocrine carcinoma**	Somatostatin analogues, 5-fluorouracil
**Squamous cell carcinoma with cervical adenopathy**	Neck dissection with possible chemotherapy and radiation therapy
**Squamous cell carcinoma with inguinal adenopathy**	Inguinal node dissection with possible chemotherapy and radiation therapy
**Tumor with CK7-/CK20+/CDX2+ molecular profile**	As per stage IV colorectal cancer
**Single metastatic site**	Local resection with possible chemotherapy and radiation therapy
**Males with bone metastases and PSA expression**	Androgen deprivation therapy with possible radiation therapy

The objective of this study was to describe how successful medical oncologists at our cancer center were in identifying the primary site, and secondly, whether or not primary site identification and adherence to current published CUP guidelines improves survival outcomes.

## Methods

### Study Design and Patient Selection

A retrospective chart review of patients with CUP, seen as a new consult by The Ottawa Hospital Cancer Centre (TOHCC) medical oncologists between January 1^st^, 2012 and September 30^th^, 2018. Inclusion criteria for study were patients referred to medical oncologist as CUP, have histological confirmed metastatic CUP, and aged 18 or above at the time of the consultation. Patients who have had another primary cancer within 5 years prior to diagnosis were excluded. The data collection protocol was approved by the Ottawa Health Science Network Research Ethics Board (OHSN-REB) with informed consent requirements waived given the retrospective nature of the study. The primary endpoint was the proportion of patients in whom an origin for the CUP was identified.

### Data Collection

Patient demographic factors were gathered from electronic medical records including age, sex, and Eastern Cooperative Oncology Group (ECOG) performance status and date of diagnosis. Initial presentation characteristics including biopsy technique, location, number of metastatic sites, and histological subtype were described. To determine which diagnosis and treatment parameters were collected, the aforementioned published ESMO and SEOM guidelines were consulted (6, 8).

Diagnostic work-up was assessed by collecting variables including complete blood count and biochemistry at presentation (hemoglobin, platelets, white blood cell count) and appropriate serum tumor markers including alpha fetoprotein (AFP), beta human chorionic gonadotropin (hCG), chromogranin A, and prostate-specific antigen (PSA) where appropriate. Immunohistochemistry (IHC) analysis of the pathology specimen including cytokeratin 7 (CK7) cytokeratin 20 (CK20), and all other tested biomarkers were recorded. Each patient was then classified for compliance as: non-compliant, primary only (completion of CK7 and CK20), partial adherence (CK7 and CK20 with at least one additional recommended IHC biomarker), and complete adherence (CK7 and CK20 with all subsequent recommended IHC biomarkers) according to the published ESMO guidelines. Imaging tests performed (*e.g.* computed tomography/CT, positron emission tomography/PET scan), genetic screening and other diagnostic procedures (e.g. endoscopy, mammography) were also recorded. Abnormal bloodwork and biochemical thresholds were set according to guidelines by the Medical Council of Canada. A primary site was considered identified only if the physician explicitly makes the diagnosis in an initial consultation or subsequent progress note.

Treatment regimens were recorded including number of treatment lines, type of treatment, treatment details (chemotherapy regimen, radiation dose and site), number of cycles, and time to first treatment from date of diagnosis. First-line treatment plans were then compared to the published ESMO and SEOM guidelines for compliance to the recommended treatment algorithm outlined as follows. Specific treatment plans were defined if a patient fell within a “favorable” subtype (**Table 1**). Patients were considered to have good prognostic status according to published guidelines if they had an ECOG 0 or 1 and normal lactose dehydrogenase (LDH). Recommended treatment for these patients included a published list of defined two-drug chemotherapy regimens (6, 8). Other patients with a poorer prognosis were recommended to receive palliative radiation, single-line chemotherapy or best supportive care (BSC). Survival parameters were collected including time to death or last follow-up from date of diagnosis, vital status and cause of death where applicable.

### Statistics

Clinicopathological factors were classified into dichotomous, categorical or continuous variables based upon clinically relevant thresholds expressed as a percentage of the cohort. Proportional differences in demographics, initial tumor presentation, date of diagnosis and diagnostic tests between the identified vs. unidentified primary experimental groups were determined using Fisher’s exact test, Chi Square test or Student’s t-test where appropriate. A univariable (UVA) Cox logistic regression analysis was used to evaluate any association between diagnostic factors and number of diagnostic tests with primary site identification. Kaplan-Meier survival curves were plotted for OS and the log-rank test used to compare differences between experimental groups. For the entire cohort, survival curves were tested and plotted for overall survival, identified vs. unidentified primary site, ECOG status 0–1 vs. 2+, and favorable subtype vs. other. In patients that did not fall within a favorable subtype but still had a favorable prognosis, survival curves were tested and plotted for those receiving treatment according to published guidelines vs. patients that did not. For all statistical analyses, the Statistical Package for Social Sciences (SPSS) version 13.0 (SPSS, Chicago, IL) and Prism 8 (GraphPad, San Diego, CA) were used. A P-value less than 0.05 was considered statistically significant.

## Results

### Patient Demographics

Patient demographics and initial tumor presentation characteristics are summarized in **Table 2**. Three hundred and five patients were identified and retrospectively reviewed after applying the outlined inclusion and exclusion criteria. The mean age was 67.8 years with males accounting for 51% of the entire cohort. One hundred and thirteen patients (37%) had ECOG PS 0-1, 62 patients (20%) had ECOG PS 2, and 130 patients (43%) had ECOG PS 3-4. The median number of metastatic sites was 2 with the liver being the most common metastatic site (48%). Median smoking pack years was 30 years. Core biopsies were obtained for majority (53%) of the cohort. Histologically, adenocarcinoma accounted for the greatest proportion of patients (*N=*163, 53%).

**Table 2:** Demographics and initial tumor presentation of entire CUP cohort.

Patient Characteristics	Total patients (%), *N=305*	Unidentified primary (%), *N=196*	Identified primary (%), *N=109*	P-value
**Age**				
Average	67.8	68.6	66.4	0.146^†^
< 39	5 (1.6)	1 (0.5)	4 (3.7)	
40–49	16 (5.2)	10 (5.1)	6 (5.5)	
50–59	54 (17.7)	36 (18.4)	18 (16.5)	
60–69	90 (29.5)	55 (28.1)	35 (32.1)	
70–79	84 (27.5)	52 (26.5)	32 (28.4)	
80+	56 (18.4)	42 (21.4)	14 (12.8)	
**Sex**				
Male	156 (51.1)	104 (53.1)	52 (47.7)	0.404
Female	149 (48.9)	92 (46.9)	57 (52.3)	
**ECOG**				
0	36 (11.8)	21 (10.7)	15 (13.8)	0.902
1	77 (25.2)	51 (26.0)	26 (23.9)	
2	62 (20.3)	36 (18.4)	26 (23.8)	
3	102 (33.4)	68 (34.7)	34 (31.2)	
4	28 (9.2)	20 (10.2)	8 (7.3)	
**Smoking Status**				
Current	57 (18.7)	34 (17.4)	23 (21.1)	0.257
Pack years (average)	37.4	34.6	42.1	
Ex	113 (37.0)	74 (37.8)	39 (35.8)	
Pack years (average)	32.3	32.0	32.8	
Never	120 (39.3)	75 (38.3)	45 (41.3)	
Unknown	15 (4.9)	13 (6.6)	2 (1.8)	
**Histological Subtype**				
Adenocarcinoma	163 (53.4)	107 (54.6)	56 (51.4)	0.214
Squamous Cell Carcinoma	38 (12.5)	25 (12.8)	13 (11.9)	
Neuroendocrine Carcinoma	47 (15.4)	35 (17.9)	12 (11.0)	
Poorly differentiated Carcinoma	34 (11.2)	17 (8.7)	17 (15.6)	
Other	20 (6.6)	10 (5.0)	10 (9.2)	
Unknown	3 (1.0)	2 (1.0)	1 (0.9)	
**Number of Metastatic Sites**				
1	104 (34.1)	71 (36.2)	33 (30.3)	0.315
2	90 (29.5)	54 (27.6)	36(33.0)	
3	64 (21.0)	45 (23.0)	19 (17.4)	
4	30 (9.8)	17 (8.7)	13 (11.9)	
5+	17 (5.6)	9 (4.6)	8 (7.3)	
**Date of Diagnosis**				
First half (2012–2015)	153 (50.2)	108 (55.1)	45 (41.3)	0.023*
Second half (2015–2018)	152 (49.8)	88 (44.9)	64 (58.7)	

^†^by Student’s t-test, all other by Fisher’s exact test or Chi-square test, * denotes statistical significance.

**Figure 1:**
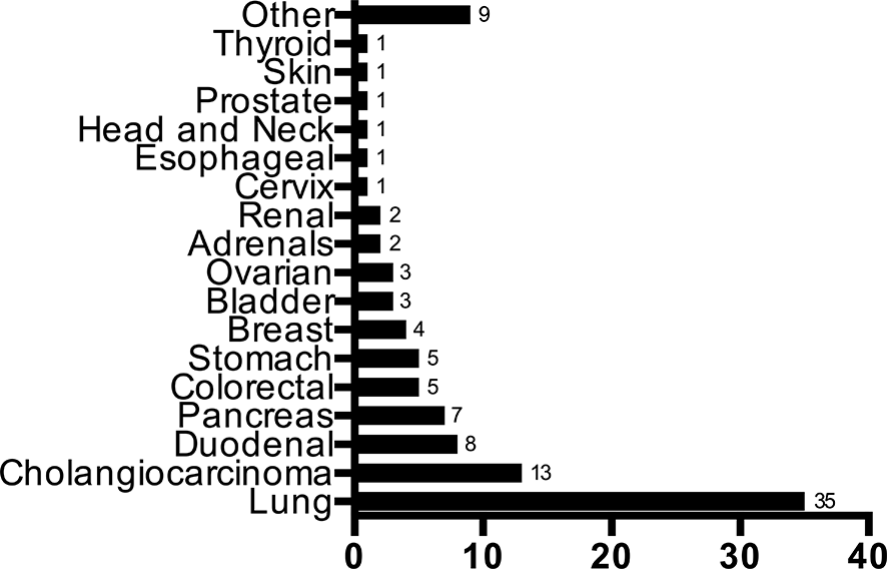
Distribution of identified primary tumour sites in 109 patients.

In our cohort, 109/305 (36%) patients had a primary identified. The distribution of the primary sites identified can be found in **Figure 1**. The most commonly predicted and identified tumor site was the lung at 35/109 (33%), followed by cholangiocarcinoma (*N=13*, 17%) and duodenal cancer (*N=8*, 7%), respectively. The majority of patients with an identified lung primary were cigarette smokers (68%). No baseline demographic or presentation factors were associated with a significantly increased proportion of primary site identification (**Tables 2**, **3**) The only significant finding was that patients diagnosed in the second half of the study (2015-2018) were more likely to have an identified primary tumor site compared to patients from the first half (42% vs. 29%; *P=0.02*).

**Table 3:** Diagnostic work-up summary of CUP cohort.

Patient Characteristics	Total patients (%), *N=305*	Unidentified primary (%), *N=196*	Identified primary (%), *N=109*	P-value
**Documented Patient History**	304 (99.7)	195 (99.5)	109 (100)	>0.999
**Documented Physical Examination**	298 (97.7)	191 (97.4)	107 (98.2)	>0.999
**Bloodwork**				
Complete Blood Count	303 (98.0)	194 (99.0)	109 (100)	0.593
Lactase Dehydrogenase (LD/LDH)	161 (52.8)	100 (51.0)	61 (56.0)	0.473
Creatinine	305 (100)	196 (100)	109 (100)	0.178
Electrolytes	299 (98.0)	191 (97.4)	108 (99.1)	0.282
Calcium	265 (86.9)	169 (86.2)	96 (88.1)	0.784
Alpha Fetoprotein (midline metastatic sites only, n=241)	58 (24.0)	35 (22.2)	23 (27.8)	0.346
Beta human chorionic gonadotropin (midline metastatic sites only, n=244)	33 (13.5)	19 (11.8)	14 (16.9)	0.324
Plasma Chromogranin A (neuroendocrine tumors only, n=51)	21 (41.2)	18 (46.2)	3 (25.0)	0.315
Prostate-Specific Antigen (male with bone metastases only, n=41)	24 (58.5)	16 (61.5)	8 (53.3)	0.719
**Immunohistochemistry (IHC)**				
CK7	229 (75.1)	144 (73.5)	85 (78.0)	
CK20	226 (74.1)	142 (72.4)	84 (77.1)	
***Adherence to published IHC diagnostic guidelines***				
**No**	89 (29.2)	59 (30.1)	30 (27.5)	
CK7 and CK20 only	65 (21.3)	47 (24.0)	18 (16.5)	
Partial adherence to additional markers	146 (47.9)	88 (44.9)	58 (53.2)	
Complete adherence to additional markers	5 (1.6)	2 (1.0)	3 (2.8)	
**Imaging/Diagnostic Tests**				
CT Head	181 (59.3)	113 (57.7)	68 (62.4)	0.467
CT Thorax	293 (96.1)	187 (95.4)	106 (97.3)	0.548
CT Abdomen/Pelvis	291 (95.4)	188 (95.9)	103 (94.5)	0.578
Octreoscan (neuroendocrine tumors only, n=49)	19 (38.8)	14 (38.9)	5 (38.5)	>0.999
Mammography (female only, n=148)	50 (33.8)	29 (31.5)	21 (37.5)	0.478
Endoscopy	121 (39.7)	80 (40.8)	41 (37.6)	0.716
Positron Emission Transmission (PET) Scan (cervical or single-site tumors only, n=117)	24 (20.5)	14 (17.9)	10 (25.6)	0.238
**Next Generation Sequencing (NGS)**	5 (1.6)	3 (1.5)	2 (1.8)	>0.999

### Diagnostic Work-Up and Identification of Unknown Primary Tumor Site

Diagnostic work-up as outlined by published guidelines can be divided into three broad categories: bloodwork, immunohistochemistry (IHC) and imaging/diagnostic tests. A summary of diagnostic work-up are outlined in **Table 3**. Almost all patients received a complete history (99.7%) and physical examination (98%). Bloodwork and biochemical compliance to guidelines was high with 98% patients testing for complete blood counts, 53% for LDH, 100% for creatinine, 98% for electrolytes and 87% for calcium. Amongst serum markers for specific patient populations, the completion rates were variable from 59% for PSA (amongst males with bone metastases) to 14% for hCG (amongst patients with midline metastases).

All published guidelines pinpoint IHC as the most important diagnostic approach for CUP. As an initial screen, testing for cytokeratin (CK) 7 and 20 can broadly triage tumors into four potential categories. CK7 and CK20 were performed for 229 (75%) and 226 (74%) patients of our entire cohort respectively while 89 patients (29%) were not evaluated for either baseline biomarker. Frequency of additional IHC markers tested that are mentioned by the published guidelines can be found in **Supplementary Table S1**. In univariate analysis, no pattern defined category was significantly associated with identification of a primary site: CK7/20 only (OR 1.1[0.6–1.9]; *P=0.64*), CK7/20 with partial adherence to additional markers (OR 1.5[0.9–2.4]; *P=0.09*), and CK7/20 with complete adherence to all additional markers (OR 2.8[0.5–16.9]; *P=0.27*).

CT thorax (96%), abdomen and pelvis (95%) were commonly done as part of the minimal basic work-up. CT scans of the head were also common amongst the entire cohort (59%) despite only being recommended for cervical metastases. Guideline compliance rates for specialized diagnostic imaging and tests where indicated are as follows: octreotide scans (39%), mammography (34%), endoscopy (40%), and PET scans (21%). Rates for next generation sequencing (NGS) completion was only 2%: only 5 patients had documented molecular testing, 4 of which were Foundation Medicine and the last patient receiving a targeted FusionPlex sequencing assay.

The average number of diagnostic tests completed was 6.30 in the identified primary cohort compared to 6.16 in the unidentified primary cohort (*P=0.44*). No threshold number of diagnostic tests was found to be significantly associated with successful identification of the primary site. Finally, in univariate analysis, no single diagnostic test was significantly associated with identification of a primary tumor site (**Table 4**).

**Table 4:** Univariable logistic regression analysis between diagnostic tests performed and primary site identification.

Diagnostic Test	Odd Ratio (95% CI)	P-value
**History and Physical Examination**	1.4 (0.3–0.9)	0.690
**Alpha fetoprotein (AFP)**	1.3 (0.7–2.5)	0.338
**Human chorionic gonadotropin (hCG)**	1.5 (0.7–3.2)	0.275
**Chromogranin A**	0.4 (0.1–1.5)	0.202
**Prostate specific antigen (PSA)**	0.9 (0.4–1.8)	0.689
**CK7/CK20 immunohistochemistry**	1.1 (0.7–1.9)	0.635
**Computed tomography (CT) Head**	1.2 (0.7–2.0)	0.420
**CT Thorax**	1.7 (0.5–7.8)	0.433
**CT Abdomen/Pelvis**	0.7 (0.2–2.3)	0.571
**Octreoscan**	1.0 (0.2–3.6)	0.978
**Mammography**	1.3 (0.6–2.6)	0.456
**Endoscopy**	0.9 (0.5–1.4)	0.584
**Positron Emission Tomography (PET) Scan**	1.5 (0.8–2.9)	0.189
**Next Generation Sequencing (NGS)**	1.2 (0.2–7.4)	0.841

### Treatment Regimen and Survival Analyses

Treatment regimens for the entire cohort are outlined in **Table 5**. Overall, 130 patients (43%) received systemic therapy and 118 patients (39%) received radiation therapy. As the first line of therapy, CUP patients received either BSC (*N=107*, 35%), chemotherapy (*N=98*, 32%), radiation therapy (*N=61*, 20%), concurrent chemoradiotherapy (*N=11*, 4%) or surgery (*N=28*, 9%). Of the 130 patients that received systemic therapy, 53 patients (41%) received only one line of treatment.

**Table 5:** Treatment summary of CUP cohort.

Patient Characteristics	Total patients (%), *N=305*	Unidentified primary (%), *N=196*	Identified primary (%), *N=109*	P-value
**First Treatment Type**				
Best Supportive Care	107 (35.1)	76 (38.8)	31 (28.4)	0.347
Chemotherapy	98 (32.1)	63 (32.1)	35 (32.1)	
Radiation Therapy	61 (20.0)	35 (17.9)	26 (23.9)	
Chemoradiotherapy	11 (3.6)	6 (3.1)	5 (4.6)	
Surgery	28 (9.2)	16 (8.2)	12 (11.0)	
Days to treatment (average)	39.7	38.9	41.0	0.649^†^
**Treatment Regimen**				
Systemic Therapy	130 (42.6)	84 (42.9)	46 (42.2)	>0.999
Radiation Therapy	118 (38.7)	69 (35.2)	49 (45.0)	0.111
**Lines of Therapy**				
1	196 (64.3)	129 (65.8)	67 (61.5)	0.850
2	62 (20.3)	37 (18.9)	25 (22.9)	
3	27 (8.9)	17 (8.7)	10 (9.2)	
4+	20 (6.6)	13 (6.6)	7 (6.4)	
**Patients receiving 2+ lines of chemotherapy**	43 (14.1)	28 (14.3)	13 (11.9)	0.604

^†^by Student’s t-test, all other by Fisher’s exact test or Chi-square test.

**Figure 2:**
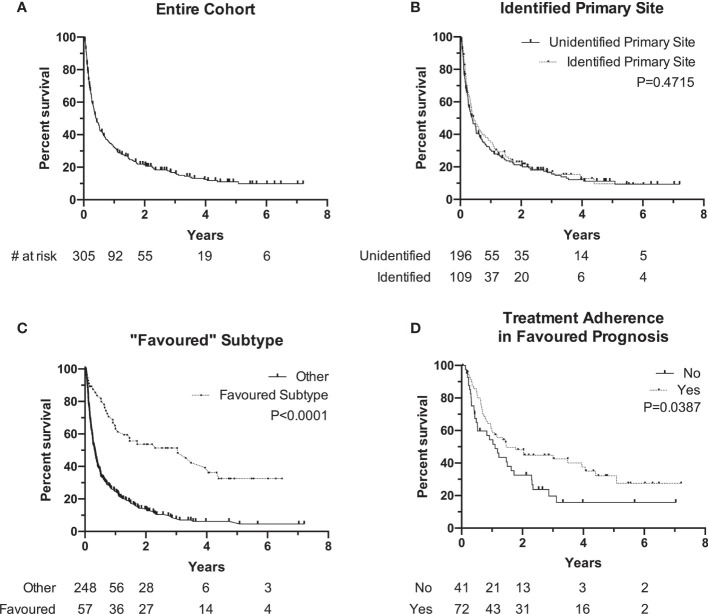
Kaplan-Meier Survival curve of overall survival (OS0 of different subgroups. **(A)** Overall cohort median OS is 4.3 months (N + 305). **(B)** OS of patients with identified primary (median, 5.2 months, N = 10 vs. unidentified primary (median, 4.2 months, N = 196). **(C)** OS of patients with "favorable" subtype (median, 36.6 months, N = 57) vs. other (median, 3.8 months, N = 248). **(D)** OS of patients with favorable prognosis (ECOG 0/1 and normal LDH) and followed traetment guidelines (median, 17.6 months, N = 72) vs. did not follow guidelines (median, 13.2 months, N = 41). Notches denote censored events.

Median OS of the entire cohort was 4.3 months [range, 0.1–86.5] with a 90-day mortality of 34% at the time this study was performed (**Figure 2A**). Cancer-related causes were responsible for 98% of patient deaths in our cohort. Survival by ECOG status only can be found in **Supplementary Figure S1** . Patients with an identified primary site did not experience a significantly longer median OS when compared to patients without an identified primary site (median, 5.2 months, identified primary vs. 4.7 months, unidentified; *P=0.47*) (**Figure 2B**). The 57 patients (19%) with a favorable subtype (**Table 1**) experienced significantly longer median OS compared to those without favorable subtype (36.6 months vs. 3.8 months, *P<0.0001*) (**Figure 2C**). Of these 57 patients, 39 (68%) were prescribed a treatment regimen consistent with the recommended published guidelines. For patients without a favorable subtype but nevertheless have a good prognostic status (ECOG 0/1 and normal LDH), those who were prescribed a two-drug chemotherapy regimen according to treatment guidelines experienced longer median OS at 17.6 months compared to their counterparts at 13.2 months (*P=0.04*) (**Figure 2D**).

## Discussion

Over the past decade, research has made significant advancements in cancer care, yet CUP remains one of the most difficult cancer types to diagnose and treat. It remains associated with a very poor prognoses as reflected by a dismal median survival of 4.3 months in our cohort (12). Clinical guidelines by several medical organizations are available to reduce the uncertainty in the patient care process by suggesting a list of diagnostic tests; however, whether Canadian oncologists comply with these guidelines is unknown. We collected diagnostic and treatment details on 305 patients referred for consultation for CUP at the Ottawa Hospital from 2012 to 2018 and compared them to clinical guidelines set out by the ESMO and SEOM. While our physicians have a high compliance rate to most diagnostic tests, only 109 (35%) of patients successfully have a primary site identified. The rationale behind wanting to identify the primary site is for several reasons. Firstly, knowing the primary site aids the clinician in choosing a more selective treatment regimen specific to that cancer type as opposed to general chemotherapy. Secondly, the primary site may inform better patient prognoses. And thirdly, knowing the cancer type can reduce the anxiety associated with uncertainty that patients with CUP face.

The proportions of demographic and initial tumor presentation characteristics between the identified primary cohort compared to the unidentified were insignificant, and no single diagnostic test was associated with predicting the primary tumor site. Both cohorts performed a similar average number of diagnostic tests. Together, we were unable to identify a specific pattern or quantity of diagnostics that are associated with increased primary site identification, rather initial tests are more essential in providing context to the histological diagnosis by the pathologist. Immunohistochemical (IHC) pathological analyses are touted by multiple guidelines to be the ultimate diagnostic test for identifying the primary site, as well as importantly excluding potentially curable tumors (lymphomas, germ-cell tumors). Cytokeratins (CK7 and CK20) are the most important initial markers to classify carcinomas and adenocarcinomas, which was followed in 71% of our patients. Additional tumor-specific biomarkers can then be performed to provide clues into the cell type and origin (6, 8). Some patterns of IHC, combined with the appropriate clinical picture, are immensely helpful in suggesting a primary site. In addition to being the most common cancer in Canada, this may explain why the lung was the most commonly identified site, accounting for 33% of all identified primary sites, consistent with descriptive analyses of CUP patients at other academic centres (13, 14). The specific IHC pattern of CK7+/CK20- with a positive thyroid transcription factor-1 (TTF1) stain was commonly encountered and was confidently diagnosed as lung cancer in almost all cases (15). Outside of a few specific IHC combinations, a systematic approach by the pathologist remains the gold-standard in reaching a specific diagnosis (16).

Of minor note, a greater proportion of patients in the second half of our study (2015–2018) had a primary site identified (42% vs. 29% from 2012 to 2015). This finding may be explained given the publication of the ESMO and SEOM CUP guidelines in 2015 and 2018 respectively, giving oncologists and pathologists a more definitive approach to finding the primary site, but assumes that those clinicians were aware of or followed those guidelines. This included an increased awareness for the standardized approach to IHC, 32% of patients did not receive CK7 and CK20 markers in the first half which modestly decreased to 26.3% in the second half (*P=0.69*).

Being on the lower spectrum of compliance, we were also particularly interested in the contribution of PET scans (performed in 21% cases) and NGS (2%) on primary site identification. Our results demonstrate that patients receiving PET scans were slightly more likely, albeit not significant, to have an identified site (18% vs. 13%; *P=0.24*). This finding is in accordance to multiple reviews highlighting that PET scans are instrumental, especially in head and neck cancers, in improving detection of primary sites missed by conventional imaging and previously undiagnosed metastases (17, 18). However, the use of PET scans is limited given the restrictions on availability and accessibility in Canada. Not all patients will be covered for PET under the Canadian provincial based healthcare system. Future studies investigating the efficacy of PET to justify its high cost remain warranted. Progress is also being made on identifying patterns in the genetic signatures of CUP in hopes of identifying patients with more responsive subtypes (19, 20). As only five patients (2%) in our study cohort underwent NGS, we are unable to make any conclusions on its efficacy in identifying the primary site or on improving OS. However, recent advances in other molecular techniques have shown promising results with a retrospective study by Moran et al. demonstrating an 87% success rate in identifying the primary tumor site by microarray DNA methylation signatures (21). It is clear that advancements in NGS is the direction that CUP diagnosis and treatment regimens should be following. Whether primary site identification by this method improves patient prognosis remains to be seen.

Our study shows that although oncologists in our academic center are consistent with CUP diagnostic guidelines in the majority of cases; however, no clear trend exists between the types of diagnostic tests performed and being able to successfully identify the primary site. Consequently, the question becomes whether a complete evaluation to identify the primary site is truly necessary. Our results would suggest against this notion as we demonstrate that despite having an identified primary site, these patients do not experience significantly improved OS compared to their unidentified counterparts (median, 5.2 months, identified vs. 4.2 months, unidentified; *P=0.47*). Rather, the main purpose of a diagnostic work-up at this point of time is to identify patients in favorable subgroups with defined treatment regimens who clearly demonstrate a superior OS (median, 36.6 months vs. 3.8 months; P<0.0001) (10). For example, the ESMO guidelines highlights a retrospective study by Hainsworth et al. demonstrating that CUP patients with an immunohistochemical profile similar to colorectal cancers responded well to colorectal-specific therapy (e.g. FOLFOX, FOLFIRI) (22).

Patients who were defined to have a favorable prognosis (not to be confused with “favorable” subtype) are recommended to undergo the recommended two-drug chemotherapy regimens to improve their prognosis (median, 17.6 months, followed guidelines vs. 13.2 months, did not follow guidelines; *P=0.04*). This finding has previously been discovered in a similar patient population (9). Patients in poorer-risk categories however, continue to be lacking in effective treatment options, with several clinical studies demonstrating no added benefit between different types of chemotherapy (23, 24). In accordance with our findings that 47% of patients without a favorable prognosis are managed with BSC, current focus should be placed on symptom management and preservation of quality of life. However, there remains hope for poor prognosis CUP patients given the positive results of the recent NivoCUP trial (UMIN000030649) (25), or our upcoming phase 2 Pembrolizumab study (NCT03391973).

The rise of immunotherapy in the recent decade will open up an entirely new discussion on its efficacy in CUP. However, given the targeted nature of immunotherapy and the ambiguous nature of CUP tumors, there needs to be a minimum level of investigation and resemblance to a particular tumor subtype in order for the chosen therapy to be effective. Immunoprofiling with biomarkers, at its current stage, has typically been unsuccessful in identifying CUP candidates for immunotherapy (26, 27). Clinical trials are ongoing investigating immunotherapy in CUP patients. As an encompassing primary site identification strategy is not yet available, future clinical trials should continue to aim in identifying CUP “favorable” subtypes that may respond well to novel therapeutic strategies. A recent breakthrough example of this by Verver et al. demonstrates that melanoma of unknown primary (MUP) are responsive to immune checkpoint inhibitors and targeted therapies, significantly improving their OS to 11 months from 4 months with these novel treatments (P<0.001) (28).

Taken together, our study did not demonstrate any particular diagnostic pattern, even suggested by published guidelines, to be superior in identifying the primary site. Rather, diagnostics should focus on identifying patients with “favorable” subtypes and prescribing chemotherapy according to published treatment guidelines. It is important to note that the conclusions drawn by our study are limited by its retrospective, single-center design. While we can identify trends of diagnosis and treatment by the oncologists at our center, the same trends may not be present in centers with different standards of care or those that have implemented different guidelines. Patients that were included in this study were first referred to medical oncology exclusively for CUP; therefore, it is possible that we were excluded patients who initially presented with CUP but had an identified primary site prior to their first consultation. In addition, our definition of an identified primary site was solely extracted from clinical notes and subject to interpretation. One oncologist might be more explicit in confirming the primary site with the same diagnostic results whereas another might not document their suspicions. Finally, the retrospective nature of this study only allows us to establish associations, not causative relationships, between our measured factors, the identification of the primary tumor site and patient outcomes. Next steps will include investigation of CUP guideline compliance at other oncology centers across the country and with this larger series, we can further evaluate whether primary site identification and guidelines adherence is truly effective for improving primary site identification and patient prognoses.

## Data Availability Statement

The raw data supporting the conclusions of this article will be made available by the authors, without undue reservation.

## Ethics Statement

The studies involving human participants were reviewed and approved by Ottawa Health Science Network Research Ethics Board (OHSN-REB). Written informed consent for participation was not required for this study in accordance with the national legislation and the institutional requirements.

## Author Contributions

BW is the primary author and was involved in protocol writing, ethics approval, study design, generation of data collection tools, collection of data, statistical analysis, interpretation of data, and writing of the manuscript. MV was involved in interpretation of study data and editing of the manuscript. PW-P is the corresponding author and was involved in protocol writing, ethics approval, study design, data collection, interpretation of data, and editing of the manuscript. All authors contributed to the article and approved the submitted version.

## Conflict of Interest

The authors declare that the research was conducted in the absence of any commercial or financial relationships that could be construed as a potential conflict of interest.
