# Supplementary material for: The Diminishing Importance of Primary Site Identification in Cancer of Unknown Primary: A Canadian Single-Center Experience
Source: Front Oncol. 2021 Mar 3;11:634563. doi: 10.3389/fonc.2021.634563 (PMC7968101; doi:10.3389/fonc.2021.634563)
Supplement: Supplementary file 1 [file DataSheet_1.docx]

Supplementary Material

**Table S1:** Proportions of additional immunohistochemical markers mentioned in the published guidelines for cancer of unknown primary set out by the European Society of Medical Oncology (ESMO) or Spanish Society of Medical Oncology (SEOM) in a cohort of 305 patients.

| Immunohistochemical marker | Number of patients received |
| --- | --- |
| TTF1 | 201 |
| CDX2 | 151 |
| PAX8 | 90 |
| ER | 89 |
| Synaptophysin | 79 |
| Chromogranin | 76 |
| PSA | 61 |
| CEA | 53 |
| WT1 | 50 |
| PR | 45 |
| AE1/3 | 37 |
| S100 | 36 |
| Napsin | 31 |
| Hep Par-1 | 23 |
| Mammaglobulin | 20 |
| Calretinin | 20 |
| Vimentin | 17 |
| HMB45 | 14 |
| Melan-A | 13 |
| CD10 | 12 |
| p63 | 10 |
| RCC | 8 |
| GCDFP15 | 7 |
| Inhibin | 6 |
| Thyroglobulin | 4 |
| AFP | 4 |
| CD13 | 3 |
| NSE | 2 |
| LCA | 2 |
| Desmin | 2 |
| CA-125 | 2 |
| CK19 | 1 |
| Calcitonin | 1 |
| PLAP | 1 |
| Mesothelin | 0 |
| Urothelin | 0 |
| CDH17 | 0 |


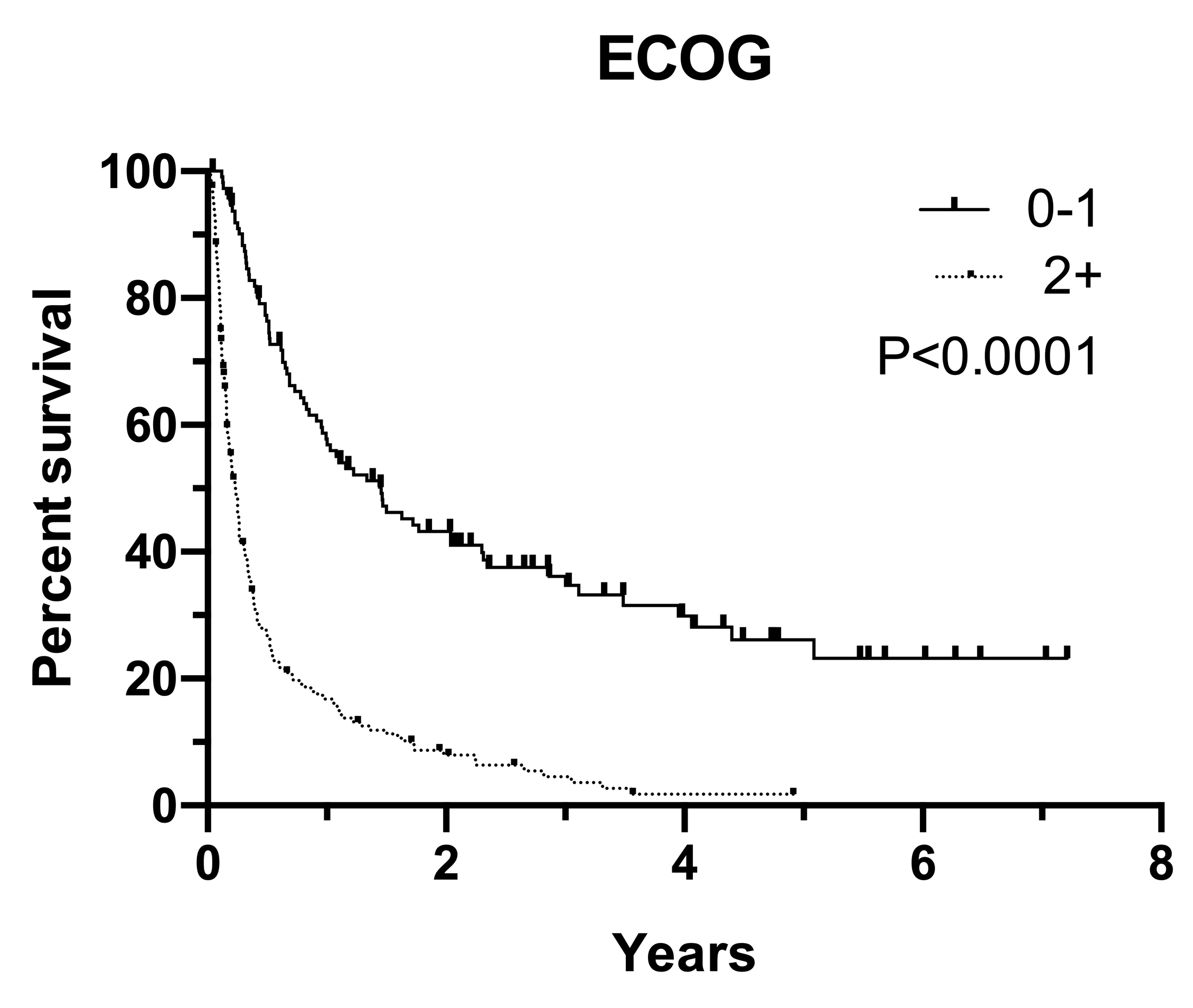


**Figure S1. Kaplan-Meier Survival curve of patients with ECOG 0/1 vs. 2+.** Overall survival of patients with ECOG status 0 or 1 (median, 17.5 months, *N=113*) vs. ECOG status 2+ (median 2.8 months, *N=192*).
